# Synthesis and performance of CMC/PAM-based interpenetrating network hydrogels for targeted urea delivery

**DOI:** 10.1039/d6ra00860g

**Published:** 2026-04-09

**Authors:** Ehab S. Gad, Saad Alrashdi, Elsayed M. Elnaggar, Ayman H. Ahmed, Medhat E. Owda

**Affiliations:** a Department of Chemistry, College of Science, Jouf University Sakaka 72341 Aljouf Saudi Arabia esgad@ju.edu.sa; b Chemistry Department, College of Science, University of Bisha Bishah 61922 Saudi Arabia; c Department of Chemistry, Faculty of Science (boys), Al-Azhar University 11884 Nasr City Cairo Egypt medhatowda@azhar.edu.eg

## Abstract

This study reports the synthesis and comprehensive characterization of carboxymethyl cellulose (CMC) and CMC/polyacrylamide (CMC/PAM) interpenetrating polymer network (IPN) hydrogels for enhanced water retention and slow nutrient release. The CMC/PAM hydrogel was fabricated *via* free-radical polymerization and systematically benchmarked against a chemically crosslinked CMC hydrogel to elucidate structure–property–release relationships. FTIR, XRD, TGA, and SEM analyses confirmed successful IPN formation, enhanced structural stability, and a more interconnected porous architecture. Compressive testing revealed significant mechanical reinforcement, with the modulus increasing from 0.10 MPa (CMC) to 0.40 MPa (CMC/PAM). Swelling studies showed that the CMC/PAM hydrogel exhibited higher water absorption capacity (46 g g^−1^) than CMC (32 g g^−1^), following Fickian diffusion behavior during the initial stage. Urea release profiles displayed biphasic behavior for both systems; however, kinetic modeling using the Higuchi, first-order, and Korsmeyer–Peppas models demonstrated a markedly reduced diffusion constant in the CMC/PAM hydrogel, confirming improved release control due to increased network density and hydrogen bonding interactions. These results demonstrate that IPN formation effectively modulates nutrient transport, supporting the classification of the developed system as a slow-release fertilizer matrix for sustainable agricultural applications.

## Abstract 2

CMC/PAM IPN hydrogels were successfully prepared by free-radical polymerization. FTIR, XRD, TGA, and SEM confirmed. Urea release kinetics establishing the hydrogel as a slow-release fertilizer matrix for agriculture applications.

## Graphical abstract

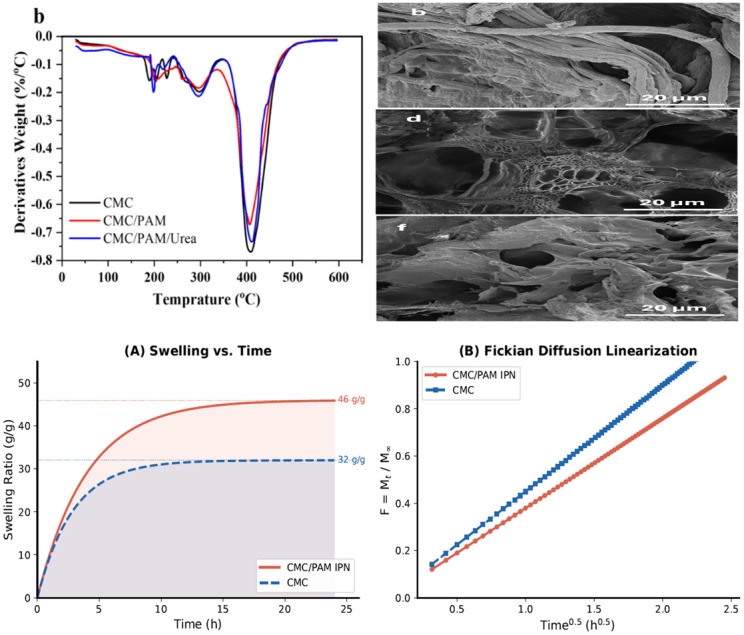

## 1 Introduction

The escalating global population, projected to increase by an additional 1.3 to 1.9 billion people between 2020 and 2050, intensifies the critical challenge of ensuring adequate food security.^1,2^ This demographic pressure coincides with diminishing agricultural land, increasingly volatile climatic conditions, and the detrimental environmental impact of conventional farming practices.^3,4^ Nitrogen (N) is an essential macronutrient vital for plant development, photosynthetic performance, and agricultural productivity. Among nitrogen-based fertilizers, urea is widely used, owing to its high nitrogen concentration and cost-effectiveness.^5,6^ However, conventional urea application suffers from significant inefficiencies due to its inherent physicochemical properties, namely, high solubility, low thermal stability, and small molecular size, which lead to substantial nitrogen losses soon after field application. These losses occur predominantly through volatilization into the atmosphere and *via* runoff or leaching into water systems.^7^ Once applied, urea is rapidly hydrolyzed by soil urease enzymes and subsequently undergoes nitrification, converting into nitrite and nitrate ions. Under excessive irrigation or heavy rainfall conditions, these ions readily leach from the soil profile, accumulating in groundwater and surface waters. This contamination poses serious environmental and public health risks. Moreover, the extensive use of urea accelerates soil acidification, depletes soil organic matter and fertility, and negatively impacts populations of beneficial soil microorganisms.^8^ This impairs the soil's ability to efficiently retain and supply nutrients, resulting in nutrient-depleted, degraded soil that undermines crop development and reduces yields. Therefore, it is essential to apply fertilizers and agrochemicals judiciously, using the minimum effective dose necessary, to prevent overuse, preserve soil fertility, and sustainably enhance agricultural productivity for farmers.^9^ An effective and increasingly favored approach to curb fertilizer overuse without compromising crop performance is the use of controlled-release or slow-release fertilizers (CRFs/SRFs). These advanced formulations can reduce fertilizer application rates by 20–30% while maintaining equivalent or even improved yields.^10,11^ Over the past two decades, SRFs have attracted growing interest in agriculture due to their potential to improve nutrient use efficiency. While “slow-release fertilizer” (SRF) is a general term describing fertilizers that gradually release nutrients over time to better match plant uptake, “controlled-release fertilizer” (slow), as precisely defined by Duan *et al.* implies a more sophisticated system capable of modulating nutrient release in response to specific plant needs or rhizosphere conditions unique to different crop species.^12^ Achieving such precision remains technically challenging. In practice, most SRFs rely on physical strategies, such as coating or encapsulated nutrients within materials that slowly dissolve or biodegrade in soil, often featuring porous structures to facilitate a consistent, prolonged release of nutrients.^13,14^ Recent hydrogel-based urea delivery systems report widely varying performance depending on polymer chemistry and crosslinking architecture. For example, a fully bio-based CNF/CMC hydrogel achieved a high-water absorption capacity of 147 g g^−1^ with urea loading up to 92 wt%, and demonstrated agronomic potential in pot testing.^15^ In contrast, nanocomposite networks engineered for long-duration delivery have demonstrated substantially extended-release times; Lu *et al.* developed LCNF-reinforced (AA-*co*-AAm) hydrogels and reported slower urea release over ∼30 days, with swelling tunable from 72 to 24 g g^−1^ depending on fiber content.^16^ CMC-based systems incorporating acrylamide chemistry have also been reported *via* different strategies, such as CMC-g-PAM graft copolymers designed as sustained/slow-release fertilizer carriers.^17^ More recently, “green” hydrogel SRF formulations have begun to include soil-relevant validation, such as column/soil release benchmarks showing 61% urea release over 42 days in soil, highlighting the importance of testing environment on release duration.^18^ Collectively, these studies show that nutrient release lifetime (days to >1 month) and swelling capacity (tens to >100 g g^−1^) depend strongly on crosslinking strategy (graft *vs.* IPN *vs.* nanocomposite) and network density. Against this background, the present work employs a CMC/PAM IPN prepared by a simple free-radical route and benchmarks its swelling and urea-release behavior directly against a CMC-only hydrogel prepared under comparable conditions, enabling clearer attribution of performance gains to IPN formation. By synchronizing nutrient availability with plant demand and minimizing losses through leaching, volatilization, or runoff, SRFs significantly enhance efficiency of the nutrient use throughout the crop growth cycle.^19^ In line with global priorities for environmental stewardship and sustainable agriculture, recent research has shifted toward developing SRFs from eco-friendly, renewable sources and biodegradable. These include agricultural residues, industrial byproducts, and even household waste materials, such as starch, natural rubber, cellulose, chitosan, gelatin, and pectin, offering a greener, circular-economy-aligned alternative to conventional synthetic fertilizers.^19^ Within the realm of advanced materials for sustainable agriculture, hydrogels stand out as particularly attractive and versatile platforms for controlled nutrient delivery. These fascinating materials are characterized as three-dimensional (3D) crosslinked polymeric networks capable of absorbing and retaining substantial amounts of water or aqueous solutions while maintaining structural integrity. Their inherent advantages, including active agent loading and sustained release, ion permeability, and biocompatibility/biodegradability, render them valuable across various biomedical and environmental applications.^20^ In the context of SRFs, hydrogels significantly reduce nutrient leaching by holding fertilizers within their polymeric matrix, protect encapsulated nutrients from rapid degradation, and ensure their extended availability to plants.^21^ Beyond nutrient release, hydrogels also critically address water scarcity, a major limiting factor in agricultural production. Their remarkable water retention capabilities are invaluable, particularly in semi-arid and arid regions, enhancing soil moisture, improving soil texture by creating more internal soil spaces for aeration and development of soil edaphon, and reducing irrigation frequency.^18,19^ Previous studies have shown that cellulose hydrogels, when mixed in soil or covering mung bean seeds, positively impacted root, stem, and leaf length by 27–410% compared to controls grown with soil only.^22^ Cellulose, the most abundant natural polymer, interacts strongly with water due to its hydroxyl (–OH) groups and is a highly suitable choice for agricultural applications as a starting material for SRFs.^19,21^ Environmentally friendly cellulose-based hydrogels are particularly important due to their non-toxic nature and low cost, which helps improve soil quality.^23^ Specifically, carboxymethyl cellulose (CMC), a significant polysaccharide resulting from the chemical modification of cellulose, is highly attractive. It is prized for its excellent biocompatibility, non-toxicity, and cost-effectiveness, and critically, the abundance of reactive hydroxyl and carboxyl (−CH_2_–COOH) functional groups along its polymeric chains.^24^ These numerous active sites provide ample opportunities for chemical crosslinking and interaction, contributing to its excellent water solubility, superior swelling capacity, and structural integrity.^23^ The incorporation of CMC into hydrogel formulations not only boosts water absorption capacity but also enhances responsiveness to pH changes, polyelectrolyte behavior, and tolerance to variations in ionic strength. Moreover, CMC modification significantly improves the hydrogel's capacity to retain essential nutrient ions, such as ammonium and nitrate, thereby reducing their leaching from the soil and promoting more efficient nutrient delivery to plants.^24,25^ In parallel, polyacrylamide (PAM), a well-established synthetic polymer, is recognized for its impressive water absorption capacity, ease of synthesis, and the presence of numerous –CONH_2_ groups.^26,27^ These amide groups facilitate various molecular interactions and crosslinking mechanisms, which not only simplify the preparation of PAM-based hydrogels but also significantly contribute to their mechanical strength and overall stability.^28^ While standalone PAM hydrogels might occasionally exhibit limitations in mechanical properties or long-term stability, the strategic combination of CMC and PAM in a composite hydrogel is envisioned to overcome these individual shortcomings.^29^ The environmental safety of PAM-based hydrogels has been critically evaluated due to concerns regarding residual acrylamide monomer toxicity and long-term soil persistence. Acrylamide monomer is classified as potentially carcinogenic; however, properly synthesized polyacrylamide hydrogels typically contain trace residual monomer levels following complete polymerization and extensive washing.^30^ In this study, prolonged purification was performed to minimize residual monomer content. While PAM itself exhibits limited biodegradability under normal soil conditions, gradual hydrolysis and microbial activity may contribute to long-term degradation.^31^ Importantly, incorporation of CMC, a biodegradable polysaccharide, enhances the environmental compatibility of the composite system. Compared with conventional polymer-coated urea fertilizers, which may generate persistent microplastic residues, hydrogel-based matrices offer a swelling-diffusion release mechanism without solid coating fragmentation.^2^

The objective of this study is to synthesize and systematically evaluate CMC and CMC/polyacrylamide (CMC/PAM) IPN hydrogels for water retention and slow-release urea delivery applications. Specifically, this work aims to (i) compare the structural, thermal, mechanical, and morphological properties of a chemically crosslinked CMC hydrogel and a CMC/PAM IPN prepared under comparable conditions; (ii) investigate their swelling behavior and diffusion mechanisms; (iii) quantify urea loading and release performance; and (iv) elucidate the relationship between network architecture and nutrient transport through kinetic modeling. By establishing a clear structure–property–release correlation, this study seeks to demonstrate how IPN formation enhances mechanical stability and regulates nutrient diffusion, supporting the development of environmentally responsible slow-release fertilizer systems.

## 2 Experimental

### 2.1. Materials and methods

Carboxymethyl cellulose (CMC, medium viscosity), acrylamide (AM, purity ≥99%), *N*,*N*′-methylenebisacrylamide (MBA), ammonium persulfate (APS), and urea (analytical reagent grade) were sourced from a certified chemical supplier and utilized as received, without additional purification. All experimental procedures were carried out using deionized water.

### 2.2. Preparation of CMC/PAM hydrogel

The CMC/PAM interpenetrating polymer network hydrogel was synthesized *via* free-radical polymerization. Briefly, 1.0 g of CMC was dissolved in 50 mL of deionized water under continuous magnetic stirring (500 rpm) at room temperature (25 ± 2 °C) until a homogeneous solution was obtained. Separately, 3.0 g of AM was dissolved in 20 mL of deionized water. The AM solution was then slowly added to the CMC solution under constant stirring to ensure uniform mixing. The total reaction volume after mixing was approximately 70 mL. Subsequently, 0.03 g of MBA (1 wt% relative to AM) was added as the crosslinking agent, followed by 0.05 g of ammonium persulfate (APS) as the initiator.^32–34^ The reaction mixture was purged with nitrogen gas at a flow rate of approximately 1 mL min^−1^ for 15 minutes to remove dissolved oxygen and minimize radical quenching. Polymerization was carried out in a thermostatically controlled water bath. The temperature of the mixture was raised to 60 °C and the reaction was allowed to proceed for 2 h. After polymerization, the obtained CMC/PAM hydrogel was immersed in excess deionized water for 48 h to remove unreacted monomers and soluble residues, with the washing water replaced every 6 h. For comparison, a CMC hydrogel was prepared separately under similar processing conditions. The purified hydrogel was then dried in a vacuum oven at 50 °C until constant weight was achieved. The dried hydrogel was ground and sieved to obtain granules with particle sizes ranging from 0.5 to 1.0 mm. For comparison, a CMC-only hydrogel was prepared by dissolving 2.0 g of CMC in 50 mL of deionized water, followed by the addition of 0.4 g of citric acid as a crosslinking agent. The mixture was heated under similar conditions to induce crosslinking. No AM, MBA, or APS were added in this formulation. The resulting gel was washed, dried, and processed using the same procedure as described for the CMC/PAM hydrogel.^35–37^

### 2.3. Urea loading

The dried hydrogels (1.0 g) of both CMC/PAM or CMC were soaked in 50 mL of a 10% w/v aqueous urea solution for 24 hours at room temperature for urea encapsulation. Following loading, the hydrogel samples were dried once more in a vacuum oven at 50 °C.

### 2.4. Characterization of the hydrogels

The dried hydrogels were thoroughly analyzed to determine their structural and thermal characteristics using a range of advanced techniques. Fourier Transform Infrared Spectroscopy (FTIR) was conducted in attenuated total reflectance (ATR) mode with a Bruker Tensor 27 spectrometer (Bruker Corp., Billerica, MA, USA), scanning from 4000 to 500 cm^−1^ at a resolution of 16 cm^−1^ and 32 scans per sample to identify chemical bonds and functional groups. Crystallinity was assessed through X-ray diffraction (XRD) using a Bruker AXS D8 diffractometer equipped with Cu-Kα radiation (*λ* = 0.154 nm), operating at 40 kV with a scanning speed of 2° per minute. The scanning was carried out over a 2*θ* range of 5° to 80°, with a scanning speed of 2° per minute. Surface morphology was examined using scanning electron microscopy (SEM) on an FEI Quanta 200 environmental SEM at an accelerating voltage of 20 kV and a working distance of 10 mm. Prior to imaging, dried hydrogel samples were fractured to expose internal cross-sections, mounted on aluminum stubs using conductive carbon tape, and sputter-coated with a thin gold layer to enhance electrical conductivity and image clarity. Thermal stability was evaluated by thermogravimetric analysis (TGA) using a Q500 analyzer (TA Instruments, Newcastle, DE, USA). Approximately 5 mg of each dried sample was heated from 30 to 600 °C at a constant heating rate of 10 °C min^−1^ under a controlled atmosphere, and the corresponding weight changes were continuously recorded as a function of temperature.

### 2.5. The degree of water swelling

To assess the water absorption capacity of the CMC and CMC/PAM hydrogel composite, the swelling ratio was measured. Dried hydrogel samples of known initial weight (denoted as *W*_0_) were submerged in distilled water at ambient temperature. At specific time intervals, the samples were retrieved, and any unabsorbed surface water was carefully removed by gently blotting with filter paper. The swollen hydrogels were then reweighed (*W*_t_), and the degree of swelling was calculated using eqn (1):1
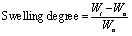
where *W*_*t*_ and *W*_o_ represent the weights of the swollen and dried hydrogel samples, respectively. Each experiment was performed in triplicate to ensure statistical accuracy.^38^

### 2.6 Swelling kinetics analysis

The water diffusion mechanism within the hydrogel network was evaluated by analyzing the initial 60% of the fractional liquid absorption data. The swelling kinetics were modeled using the following eqn (2) and (3):2
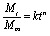


*M*_*t*_ represents the mass of water absorbed at time *t*, while *M*_∞_ denotes the water uptake at equilibrium. The parameter *k* is the kinetic rate constant, and *n* is the diffusion exponent, which characterizes the type of transport mechanism governing water absorption.

### Loading and release of urea

To achieve optimal urea absorption, pre-weighed dried hydrogel samples (*m*_0_, g) were immersed in aqueous urea solutions with concentrations ranging from 0.5 to 2.0 g L^−1^ and allowed to equilibrate for 24 hours at 25 ± 2 °C. After loading, the swollen hydrogels were removed, gently blotted to eliminate excess surface solution, and dried in a vacuum oven at 40 °C until a constant weight (*m*_1_, g) was reached.

The urea loading capacity (LC, %) was calculated using the following equation:

To achieve optimal urea absorption, pre-weighed dried hydrogel samples were submerged in aqueous urea solutions with concentrations ranging from 0.5 to 2.0 g L^−1^ and left to soak for 24 hours at ambient temperature. Following this loading process, the hydrogels, now swollen, were placed in a vacuum oven set at 40 °C and dried until their weight stabilized. The amount of urea incorporated into the CMC/PAM hydrogel composite was then quantified using was calculated using eqn (3):3
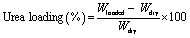
*W*_dry_ and *W*_loaded_ denote the masses of the hydrogel before and after urea loading, respectively.

To assess the urea release kinetics, a precisely weighed amount of urea-loaded hydrogel was placed in 100 mL of deionized water at ambient temperature under static (non-agitated) conditions. At scheduled time points, samples of the release medium were collected and immediately replenished with an equivalent volume of fresh deionized water to ensure consistent volume and sink conditions throughout the experiment. The presence and concentration of urea in the samples were determined spectrophotometrically at 440 nm using *p*-dimethylaminobenzaldehyde as the colorimetric reagent.^28,39^ Each experiment was performed in quintuplicate to ensure reproducibility and statistical reliability.

## 3 Results and discussion

### 3.1. Structural and thermal characterization of hydrogels

**Fig. 1:**
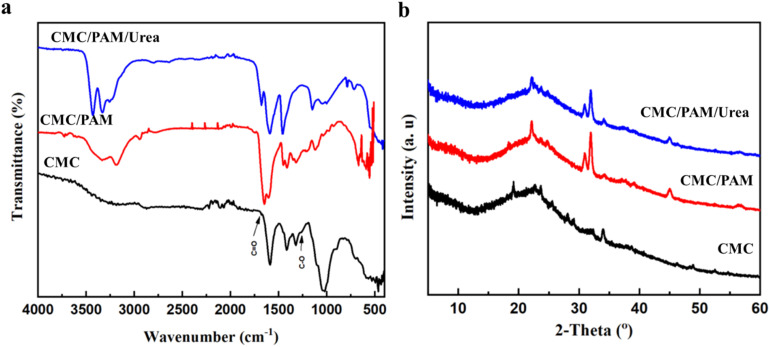
(a) FTIR (b) XRD spectra of CMC, CMC/PAM and CMC/PAM/urea hydrogel.

To elucidate the chemical structure, crystallinity, and thermal stability of the prepared hydrogels, FTIR, XRD, and TGA analyses were performed. The FTIR spectra of the CMC, CMC/PAM, and CMC/PAM/Urea hydrogels (Fig. 1a) confirm successful network formation and urea incorporation. For CMC hydrogel, a broad absorption band around 3400 cm^−1^ corresponds to –OH stretching vibrations of the hydroxyl groups, while the band near 1600 cm^−1^ represents asymmetric stretching of the –COO^−^ groups from carboxymethyl substitution.^29^

These spectral features are characteristic of cellulose-based derivatives.^30,31^ Moreover, the absorption peak at 1250 cm^−1^ is attributed to the C–O functional group, and the peak at 1677 cm^−1^ is assigned to the carbonyl (C=O) group. The observation of the carbonyl band supports the occurrence of crosslinking.^35^

Upon forming the CMC/PAM hydrogel, significant spectral changes are observed. A distinct amide I peak near 1650 cm^−1^ and an amide II peak near 1450 cm^−1^ confirm the incorporation of polyacrylamide chains, indicating the successful polymerization of acrylamide and formation of an interpenetrating polymer network.^2^ The increased intensity and slight shift of the –OH peak suggest hydrogen bonding interactions between the –OH and –COOH groups of CMC and the –CONH_2_ groups of PAM, strengthening the hydrogel network.^40^ The CMC/PAM loaded urea hydrogel, additional bands emerge around 1460–1470 cm^−1^ and 1670–1680 cm^−1^, corresponding to N–H bending and C=O stretching vibrations of urea.^41^ The broadening of the –OH and amide regions in this sample further indicates strong hydrogen bonding interactions between urea molecules and the polymeric network. XRD patterns of the CMC, CMC/PAM, and CMC/PAM/urea hydrogels (Fig. 1b) reveal significant changes in crystallinity and structural organization resulting from hybridization and urea incorporation. The CMC hydrogel exhibits a broad, less intense peak around 2*θ* = 20°, characteristic of its semi-crystalline nature, which is mainly due to the amorphous regions in the cellulose backbone with partial ordered arrangements.^29^ Upon forming the CMC/PAM hydrogel, the XRD pattern shows more pronounced peaks at approximately 2*θ* = 30–32°and 45° indicating that the introduction of polyacrylamide chains led to enhanced network ordering and partial increasing short-range structural ordering within the interpenetrating polymer matrix.^2,17^ This structural rearrangement is attributed to strong intermolecular interactions, such as hydrogen bonding, between the hydroxyl and carboxyl groups of CMC and the amide groups of PAM, which stabilize the hydrogel matrix. In the case of the CMC/PAM hydrogel loaded urea hydrogel, the XRD spectrum exhibits even sharper and more intense peaks compared to the CMC/PAM system.^33^ This enhancement in crystallinity suggests that urea molecules interact within the polymer network, potentially forming additional hydrogen bonds and promoting a tighter, more ordered arrangement of polymer chains. Such interactions may also contribute to the improved thermal stability observed in the TGA results, as the denser and more crystalline network provides stronger resistance to thermal and mechanical stress.

**Fig. 2:**
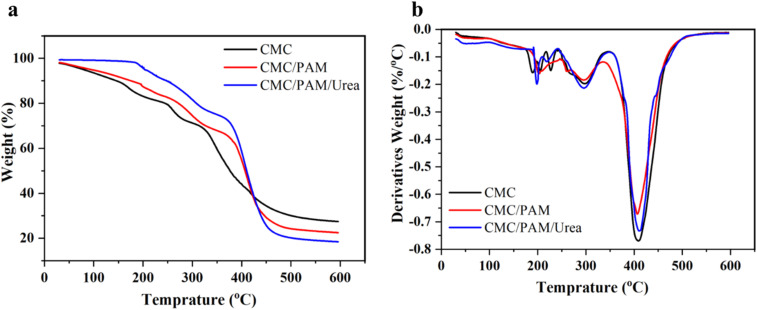
(a) The TGA (b) and DTG curves of CMC, CMC/PAM and CMC/PAM/urea hydrogel.

The thermal stability and decomposition characteristics of the CMC, CMC/PAM, and CMC/PAM/Urea hydrogels are revealed through their thermogravimetric analysis (TGA) and derivative thermogravimetric (DTG) profiles, as illustrated in Fig. 2a and b. These curves offer critical information on the thermal degradation patterns and relative stability of each hydrogel formulation. All samples exhibited an initial minor weight loss below 150 °C, which can be attributed to the removal of physically adsorbed moisture and loosely bound water molecules within the hydrogel networks. The primary decomposition phase occurred between 250 °C and 450 °C, corresponding to the breakdown of the polymer backbones of CMC and PAM.^2^ Among the three samples, the CMC showed the earliest onset of degradation and the highest mass loss rate, indicating lower intrinsic thermal stability. CMC's weight loss is caused by the decarboxylation of carboxylic groups in its structure.^38^ In contrast, the CMC/PAM hydrogel demonstrated a noticeable shift toward higher decomposition temperatures, suggesting that the interpenetrating polymer network and enhanced crosslinking between CMC and PAM chains provided improved structural rigidity and resistance to thermal degradation. The CMC/PAM/urea hydrogel showed a similar thermal pattern to the CMC/PAM matrix but with a slightly delayed main degradation peak, indicating that urea incorporation did not compromise the structural stability of the hybrid hydrogel. Instead, the interaction between urea molecules and the polymer network likely contributed to additional stabilization effects, possibly by hydrogen bonding with the –OH and –CONH_2_ groups in the matrix. Beyond 450 °C, the gradual weight loss in all samples corresponds to the decomposition of carbonaceous residues, with the CMC/PAM/Urea hydrogel retaining the highest residual mass, highlighting its superior char-forming ability and enhanced thermal endurance.^41^

The thermal degradation parameters derived from TGA and DTG analyses are summarized in Table 1. The onset degradation temperature (*T*_onset_), defined as the temperature corresponding to 5% weight loss, increased from 107.46 °C for CMC to 128.21 °C for CMC/PAM and further to 210.58 °C for CMC/PAM/urea, indicating improved initial thermal resistance in the modified systems. The maximum degradation temperature (*T*_max_), obtained from the DTG peak, occurred at 256.48 °C for CMC, while CMC/PAM and CMC/PAM/Urea exhibited *T*_max_ values of 196.50 °C and 201.58 °C, respectively. Although minor shifts in *T*_max_ were observed, these variations should be interpreted cautiously, as hydrogels are predominantly amorphous systems and small temperature differences may fall within experimental uncertainty. The residual mass at 600 °C were found to be 27.42, 22.47, 18.42% for CMC, CMC/PAM and CMC/PAM/urea respectively, suggesting differences in char formation behavior associated with network composition and urea incorporation.

**Table 1:** Thermal degradation parameters of CMC, CMC/PAM, and CMC/PAM/urea hydrogels

Samples	*T*_onset_ (°C)	*T*_max_ (°C)	Residual mass at 600 °C (%)
CMC	107.46	256.48	27.42
CMC/PAM	128.21	196.50	22.47
CMC/PAM/urea	210.58	201.58	18.42

### 3.2. Morphological characterization

**Fig. 3:**
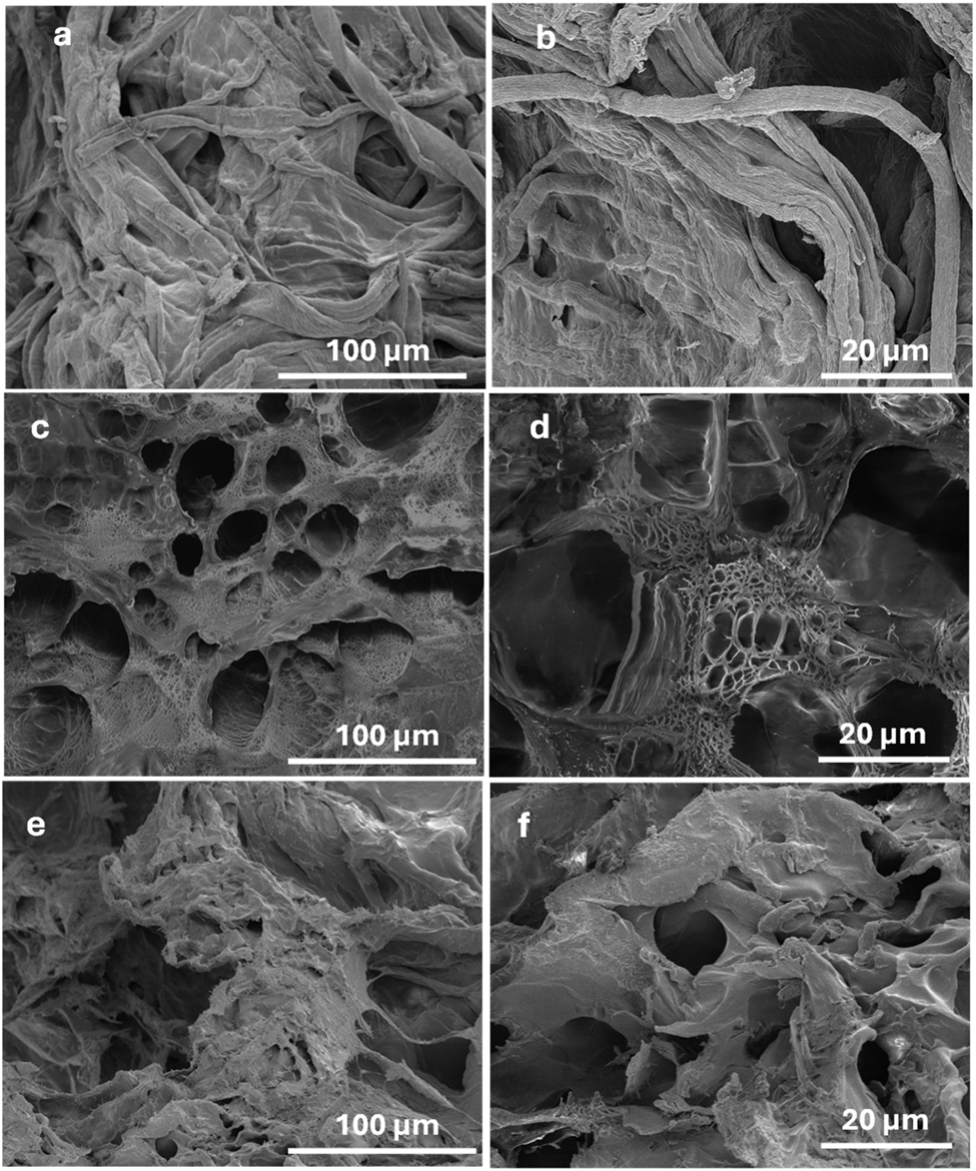
The SEM micrographs of CMC (a and b) CMC/PAM (c and d) and CMC/PAM/urea hydrogel (e and f).

The scanning electron microscopy (SEM) images (Fig. 3) provide detailed insights into the microstructural characteristics of the CMC, CMC/PAM, and CMC/PAM/urea hydrogels. For the CMC (Fig. a and b), the surface exhibits a relatively dense and compact structure with limited porosity.^42^ The polymer chains appear closely packed with fewer interconnected pores, which may restrict water uptake and limit the hydrogel's swelling ability. This morphology aligns with the lower water absorption and faster urea release observed in the CMC, as the compact structure offers less space for water and nutrient entrapment. In the CMC/PAM hydrogel (Fig. c and d), the network becomes highly porous with interconnected pores of varying sizes, indicating the successful formation of an interpenetrating polymer network between CMC and PAM chains. These well-defined pores enhance the hydrogel's ability to absorb water and retain nutrients by providing increased internal surface area and diffusion pathways. This structural feature is consistent with the higher swelling ratios and controlled urea release behavior demonstrated in the swelling and release studies. For the CMC/PAM/urea hydrogel (Fig. e and f), the porous network remains evident but appears slightly denser and more uniform compared to the CMC/PAM hydrogel. This indicates that urea loading partially occupies the pore spaces while maintaining an interconnected structure. The interaction of urea molecules with the polymer matrix likely contributes to the observed enhancement in thermal stability and sustained nutrient release, as the encapsulated urea diffuses more gradually through the denser polymer network.

**Fig. 4:**
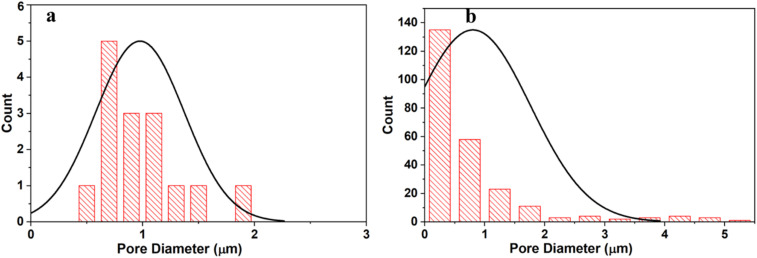
Pore size distribution of (a) CMC and (b) CMC/PAM hydrogels.

The pore size distribution obtained from SEM images using ImageJ reveals clear structural differences between the CMC and CMC/PAM hydrogels. Fig. 4a CMC hydrogel shows a relatively narrow and uniform pore size distribution, mainly centered around ∼1 µm. The limited presence of larger pores indicates a compact and less interconnected network. This dense structure restricts water diffusion pathways, which explains the lower swelling capacity and the faster urea release behavior observed for CMC. Fig. 4b CMC/PAM hydrogel exhibits a broader and right-skewed pore size distribution, with a higher number of small pores and some larger interconnected pores. The formation of the interpenetrating polymer network (IPN) increases structural heterogeneity and pore interconnectivity. This porous architecture enhances water absorption and slows nutrient diffusion, resulting in improved swelling performance and more controlled urea release.

### 3.3. Compressive mechanical properties of CMC and CMC/PAM hydrogels

**Fig. 5:**
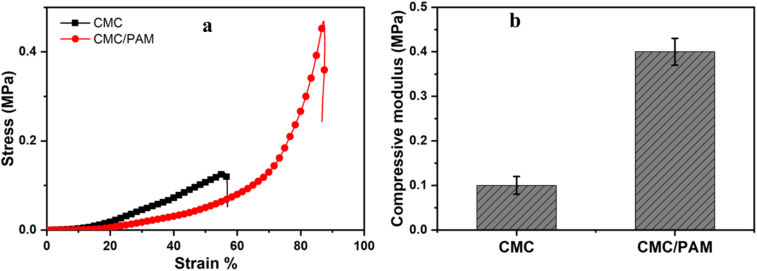
Mechanical properties of CMC and CMC/PAM hydrogels: (a) compressive stress–strain curves (b) corresponding compressive modulus values.

The compressive stress–strain curves (Fig. 5) show typical nonlinear hydrogel behavior, with a low-stress elastic region at small strains followed by pronounced strain-hardening at higher deformation. The CMC hydrogel fractured at ∼55–60% strain with a maximum stress of ∼0.12 MPa, indicating a comparatively weak and brittle network. By contrast, the CMC/PAM hydrogel sustained much larger deformation (∼85–90% strain) and reached a markedly higher maximum compressive stress (∼0.45 MPa), confirming that PAM incorporation substantially reinforces the network through IPN formation and additional physical/chemical junctions (*e.g.*, chain entanglement and hydrogen bonding between CMC –OH/–COO^−^ groups and PAM –CONH_2_ groups). This strength enhancement is consistent with prior CMC/PAM IPN reports: Jeong *et al.* reported that dual-crosslinked CMC/PAM IPN hydrogels exhibited compressive strength five times higher than CMC gels.^34^ Brusentsev *et al.* reported a maximum compressive stress of 0.45 MPa at 68% strain for a CNF-based photocrosslinked hydrogel.^43^ Viboonratanasri *et al.* reported that PAAm/CMC hydrogels exhibit a wide range of compressive fracture stresses, with CMC hydrogel showing very low values (∼0.02 MPa), while significantly higher strengths are achieved upon increasing PAAm content and optimizing network architecture.^44^ The compressive modulus further confirms this reinforcement effect, increasing from approximately 0.10 MPa for CMC to 0.40 MPa for CMC/PAM, indicating a substantial enhancement in network stiffness due to IPN formation.

### 3.4. Water absorption and swelling kinetics

**Fig. 6:**
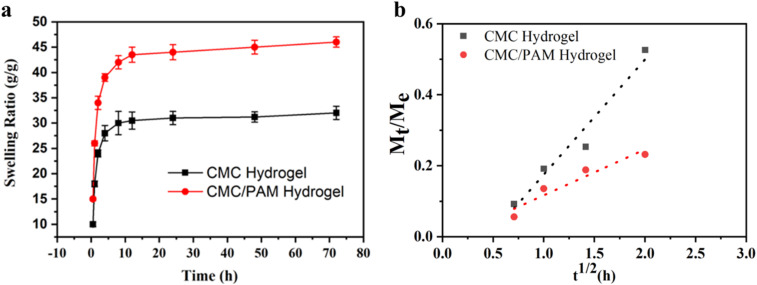
(a) Swelling ratio of CMC and CMC/PAM hydrogels over time in deionized water. (b) Linear fitting of *M*_*t*_/*M*_∞_*versus t*^1/2^ (h) during the initial swelling stage for CMC and CMC/PAM hydrogels, indicating Fickian-controlled diffusion.

The swelling behavior of hydrogels is a critical parameter for evaluating their water retention capacity and network structure, which directly influences their potential as slow-release fertilizer carriers. As shown in Fig. 6a, both the CMC and CMC/PAM hydrogels exhibited a rapid water uptake in the initial hours (0–4 h), followed by a slower increase until equilibrium was reached. However, the CMC/PAM hydrogel demonstrated a consistently higher swelling ratio, achieving a maximum of 46 g g^−1^ compared to 32 g g^−1^ for the CMC hydrogel. This enhanced water uptake is attributed to the interpenetrating polymer network (IPN) formed by CMC and PAM, which increases the density of hydrophilic functional groups (–OH, –COOH, and –CONH_2_) and generates a more porous and flexible structure for water diffusion and retention. The improved swelling of the CMC/PAM hydrogel is consistent with its porous morphology observed in SEM analysis (Fig. 3), where interconnected pores provide increased internal surface area and facilitate more efficient water absorption. This structural modification also helps the hydrogel maintain stability during the swelling process, preventing network collapse and enabling repeated cycles of swelling and deswelling without significant loss of integrity.

The initial 60% of the fractional swelling data was fitted to the Fickian diffusion model (Fig. 6b), and the corresponding parameters are summarized in Table 2. Both hydrogels exhibited *n* values below 0.5 (0.250 for CMC and 0.215 for CMC/PAM), confirming that the swelling mechanism during the early stage is primarily governed by Fickian diffusion, where water transport is controlled by molecular diffusion rather than polymer relaxation. Interestingly, the diffusion constant (*k*) was slightly higher for the CMC/PAM hydrogel (0.253) compared to CMC (0.221), indicating a marginally faster initial water penetration despite its denser crosslinked network. This behavior can be attributed to the presence of additional amide groups in PAM, which enhance water affinity and facilitate molecular diffusion through hydrogen bonding. The lower *n* value for CMC/PAM suggests a more uniform and controlled swelling profile, which is advantageous for maintaining structural integrity and ensuring steady water release in soil environments. The strong correlation coefficients (*R*^2^ = 0.9586 for CMC and 0.9186 for CMC/PAM) confirm the suitability of the Fickian diffusion model to describe the swelling kinetics of both systems. These results align with previous studies on cellulose- and PAM-based hydrogels, which reported similar values and highlighted the role of polymer composition and crosslinking density in dictating water absorption behavior.^37,39^

**Table 2:** Fickian diffusion parameters

Sample	*k*	*n*	*R* ^2^
CMC hydrogel	0.221	0.250	0.9586
CMC/PAM hydrogel	0.253	0.215	0.9186

**Fig. 7:**
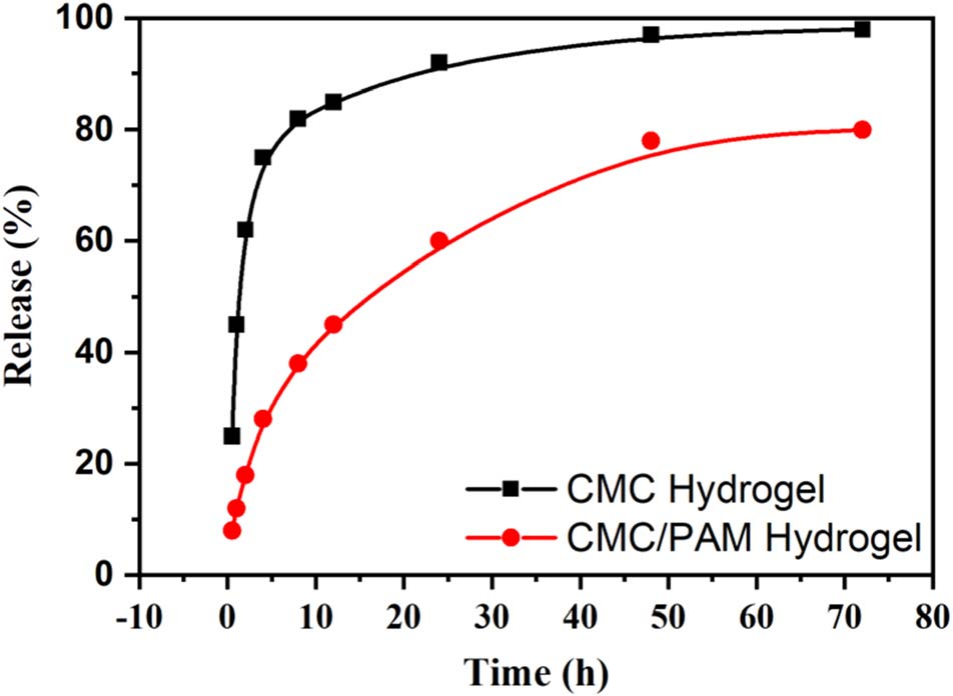
Cumulative urea release profile of CMC and CMC/PAM hydrogels in deionized water over time.

The urea release presented in Fig. 7 reveals clear differences in the release kinetics between the CMC and CMC/PAM hydrogels. Both systems exhibit a biphasic release pattern, characterized by an initial burst release during the first 12–24 hours, followed by a slower and more controlled release phase extending beyond 7 days. This initial burst is attributed to the rapid diffusion of urea molecules located on or near the hydrogel surface and in the loosely bound pores of the polymer network. However, the CMC/PAM hydrogel demonstrated a significantly slower and more controlled release profile compared to the CMC-only hydrogel. By the end of the testing period, the CMC hydrogel released more than 85% of its loaded urea, while the CMC/PAM hydrogel released approximately 70–75%. This controlled release behavior is primarily due to the interpenetrating polymer network formed by the integration of PAM, which enhances network density and creates narrower diffusion pathways for urea migration. The additional hydrogen bonding interactions between urea and the amide groups in PAM likely contribute to increased retention and delayed release, making the CMC/PAM hydrogel more efficient for sustained nutrient delivery. This characteristic offers significant benefits in agricultural settings by minimizing the loss of nutrients through leaching and maintaining a consistent release of nitrogen to crops over time. As a result, it enhances the effectiveness of fertilizers while also mitigating potential environmental hazards. Similar results have been reported in cellulose-based and IPN hydrogel systems used for controlled-release fertilizers.^43,44^

#### 3.4.1 Urea release kinetics

To better understand the release mechanism of urea from CMC and CMC/PAM hydrogels, the experimental release data were fitted to three commonly used kinetic models: the Higuchi model, the first-order model, and the Korsmeyer–Peppas model.^45–48^

The Higuchi model describes diffusion-controlled release from a matrix system and is expressed as:4
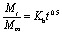
where = *M*_*t*_ amount of urea released at time *t*, *M*_∞_ = total amount released at equilibrium, *k*_H_ = Higuchi release constant and *t* = time (h)

The first-order kinetic model assumes release rate is concentration-dependent and is expressed as:5
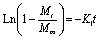
where *K*_1_ equal first-order release constant.

The semi-empirical Korsmeyer–Peppas model is used to determine the release mechanism:6
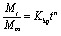
where *K*_kp_ equal the kinetic constant and *n* = diffusion exponent indicating mechanism.

**Fig. 8:**
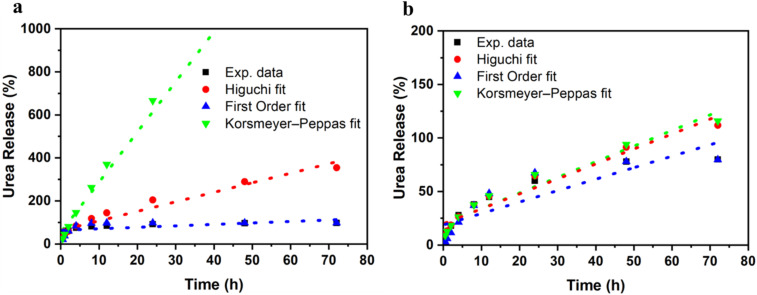
Kinetic modeling of cumulative urea release from (a) CMC and (b) CMC/PAM hydrogels.

The cumulative urea release profiles of CMC and CMC/PAM hydrogels were fitted using the Higuchi, first-order, and Korsmeyer–Peppas models, and the corresponding kinetic parameters are summarized in Table 3. Fig. 8 illustrates the comparative fitting of the experimental data with each model. For the CMC hydrogel, the Higuchi model exhibited the highest correlation coefficient (*R*^2^ = 0.9963), confirming that urea release is predominantly governed by diffusion through the hydrated polymer matrix. The high Higuchi constant (*k*_H_ = 41.79%·h^−1/2^) indicates rapid diffusion, consistent with the loose and highly swollen structure of CMC hydrogel. Although the first-order model also provided acceptable agreement (*R*^2^ = 0.9417; *k*_1_ = 0.4761 h^−1^), its predictive capability was lower than that of the diffusion-based Higuchi model. The Peppas model showed poor fitting for CMC (*R*^2^ = 0.3248), indicating that simple power-law behavior does not adequately describe the full release process. In contrast, the CMC/PAM interpenetrating polymer network hydrogel demonstrated moderated release behavior. The Higuchi model again provided strong correlation (*R*^2^ = 0.9508), confirming diffusion-controlled release. However, the significantly lower Higuchi constant (*k*_H_ = 13.18%·h^−1^/^2^) compared to CMC clearly indicates reduced diffusion rate due to increased network density. The first-order model showed comparable agreement (*R*^2^ = 0.9417; *k*_1_ = 0.07723 h^−1^), suggesting that concentration-dependent release also contributes. The Peppas exponent for CMC/PAM (*n* = 0.5149) indicates predominantly Fickian diffusion, whereas the higher exponent for CMC (*n* = 0.8480) reflects anomalous transport influenced by polymer relaxation.

**Table 3:** Kinetic parameters for urea release from CMC and CMC/PAM hydrogels

Sample	Model	*k*_H_ (%·h^−1/2^)	*k*_1_ (h^−1^)	*k*_KP_	*n*	*R* ^2^
CMC	Higuchi	41.79	—	—	—	0.9963
	First-order	—	0.4761	—	—	0.9417
	Korsmeyer–Peppas	—	—	45.00	0.8480	0.3248
CMC/PAM	Higuchi	13.18	—	—	—	0.9508
	First-order	—	0.07723	—	—	0.9417
	Korsmeyer–Peppas	—	—	12.81	0.5149	0.7649

## 4 Conclusion

This study successfully developed and evaluated CMC and CMC/PAM interpenetrating polymer network (IPN) hydrogels for potential applications in water retention and controlled urea release. The incorporation of PAM into the CMC matrix significantly enhanced the hydrogel's properties, including higher water absorption capacity (∼46 g g^−1^*vs.* ∼32 g g^−1^ for CMC), more controlled swelling kinetics, and improved thermal and structural stability. Kinetic modeling confirmed that the swelling of both hydrogels followed Fickian diffusion during the initial phase, with pseudo-second-order kinetics providing the best fit overall, indicating that swelling is driven by water–polymer interactions and network relaxation. The CMC/PAM hydrogel also demonstrated superior controlled-release performance, reducing the initial burst release of urea and enabling sustained nutrient delivery over time. These results suggest that the CMC/PAM hydrogel system is a promising candidate for agricultural applications, offering enhanced water retention, improved nutrient use efficiency, and reduced environmental losses. Future work could optimize the network composition or integrate functional additives to further tailor the hydrogel's performance for specific soil and crop conditions.

## Author contributions

Ehab S. Gad: formal analysis, investigation, methodology, writing – review & editing data curation. Saad Alrashdi, Elsayed M. Elnaggar and Ayman H. Ahmed: writing – review & editing – data curation. Medhat E. Owda: conceptualization, formal analysis, investigation, methodology, writing – review & editing data curation.

## Conflicts of interest

The authors declare that this manuscript was prepared in the absence of any personal, financial, or non-financial conflicts of interest.

## Data Availability

The complete set of data generated and analyzed in this study is fully presented within this published article.
